# The efficacy and adverse events of mTOR inhibitors in lymphangioleiomyomatosis: systematic review and meta-analysis

**DOI:** 10.1186/s13023-018-0874-7

**Published:** 2018-08-14

**Authors:** Nannan Gao, Tengyue Zhang, Jiadong Ji, Kai-Feng Xu, Xinlun Tian

**Affiliations:** 1 Department of Respiratory Medicine, Peking Union Medical College Hospital, Peking Union Medical College, Chinese Academy of Medical Sciences, Beijing, China; 2 School of Statistics, Shandong University of Finance and Economics, Jinan, China

**Keywords:** Lymphangioleiomyomatosis, mTOR inhibitors, Meta-analysis

## Abstract

**Background:**

Lymphangioleiomyomatosis (LAM) is a rare lung disease and the mammalian target of the rapamycin (mTOR) inhibitors has been used as an effective therapy. Here we conducted a systematic review and meta-analysis with the aims to quantify the efficacy and safety of mTOR inhibitors in LAM patients.

**Methods:**

The following databases were searched for clinical trials regarding LAM patients treated with mTOR inhibitors until December 2017: Pubmed, Embase, Cochrane Library and OVID medicine. Random effect models were used for the quantitative analysis.

**Results:**

Nine eligible studies were included in our systematic review, 7 of which were used for the meta-analysis. In LAM patients, mTOR inhibitors improved forced expiratory volume in 1 s (FEV_1_) and forced vital capacity (FVC) significantly, with the weighted mean difference (WMD) 0.15 L (95%CI: 0.08 to 0.22, *P* < 0.01, *I*^*2*^ = 0%) and 0.22 L (95%: 0.11 to 0.32, *P* < 0.01, *I*^*2*^ = 0%) respectively. There was no significant change in neither the diffusing capacity for carbon monoxide (WMD: 0.51 ml/mm Hg/min, 95%CI: -0.48 to 1.49, *P* = 0.31, *I*^*2*^ = 0%) nor 6-min walking distance (WMD: 5.29 m, 95%CI: -18.01 to 28.59, *P* = 0.66, *I*^*2*^ = 1%). The weighted partial response rate was 0.68 (95%CI: 0.53 to 0.84, *P* < 0.01, *I*^*2*^ = 72%) for renal angiomylipoma. The cumulative incidence rates of common safety events were 50, 40, 23, 20 and 19% for oral mucositis, hyperlipidemia, headache, bone marrow suppression, and diarrhea, respectively. And most events were low grade and tolerant.

**Conclusions:**

In LAM patients, there are improvements of FEV_1_ and FVC after the application of mTOR inhibitors and over a half achieved the shrinkage of renal angiomyolipoma.

**Trial registration:**

PROSPERO registration number: CRD42018085470. Registered 22 January 2018.

**Electronic supplementary material:**

The online version of this article (10.1186/s13023-018-0874-7) contains supplementary material, which is available to authorized users.

## Background

Lymphangiomyomatosis (LAM), characterized by progressive cystic destruction of lungs, recurrent pneumothorax, chylothorax and abdominal tumors, is a rare low-grade systemic neoplasm, exclusively affecting women [1–3]. LAM lesions are generated by the proliferation of LAM cells. The mutations of either *TSC*1 or *TSC2* gene activate the mammalian target of rapamycin (mTOR) signaling pathway, resulting in cellular aberrant function and tumor growth [4, 5]. Although with smooth-muscle feature and benign appearance [6], the LAM cells carrying inactivated *TSC* gene can migrate in blood and lymphatic fluids to form lesions in other organs [7].

LAM occurs sporadically (sLAM) with an incidence of 5 per million women [8] or associated with tuberous sclerosis complex (TSC-LAM), affecting 30–40% TSC women patients [9]. Patients may be asymptomatic in early stages. However, the LAM patients often present varied clinical manifestations with respiratory symptoms, lymphatic masses, chylous complications and severe intratumoral hemorrhage of renal angiomyolipoma. In the progressive patients, the pulmonary function declines by 2–4 folds than normal age-related decline rate or even more, the lung transplantation is the only option for the patients at the end-stage [10, 11].

Hopefully, mTOR inhibitors have shown the significant therapeutic effects on the regression of angiomyolipoma volume and the partial improvement or stabilization on the lung function in TSC or LAM patients [12–14]. As one agent of the mTOR inhibitors, Sirolimus has been recommended as the standard therapy for LAM patients with declining lung function and problematic chylous effusions [15]. Despite of these benefits, as an immunosuppressor, sirolimus has raised concerns for its adverse events, correlated with the dose or the therapy duration. However, LAM is an orphan disease, which inevitably limits the sample size in clinical trials, making it difficult to design studies or to integrate variable data. So far, there is no quantitative analysis evaluating the therapeutic efficacy and adverse events for the therapy.

Herein, based on the published and extension results, we conduct the literature review to present the updated insights on the therapy. Furthermore, we try to support the quantitative evidence to assess the efficacy and adverse events for LAM patients treated with mTOR inhibitors.

## Methods

### Information sources and search strategy

Studies were identified by searching Pubmed, Embase, Cochrane Library and OVID medicine until 31 December 2017. The search strategy included the following term keys: (‘lymphangioleiomyomatosis’) AND (‘mTOR inhibitor’ OR ‘rapamycin’ OR ‘sirolimus’ OR ‘everolimus’). Publications were restricted in humans and English and Chinese language during the literature search. Study types were limited to clinical trial, meta-analysis, randomized controlled trial or review. In addition, we reviewed the references of included articles as a complement of the related articles not inclusive in the initial search. We also contacted authors of relevant papers regarding further published and unpublished work. This meta-analysis was registered in the PROSPERO database with the registration number CRD42018085470.

### Study selection

Two reviewers (NG and TZ) independently performed the initial search and the eligibility assessment. NG and TZ respectively screened out the related studies through titles and abstracts of all articles. Disagreements were resolved by consensus between all authors. Then full text articles were assessed for eligibility by the authors.

### Inclusion and exclusion criteria

Original articles were included if they met the inclusion criteria: (1) any phase clinical trial evaluating the mTOR inhibitors on sLAM or TSC-LAM, whether they had control groups or not; (2) efficacy and safety data could be found in the full text. Exclusion criteria were as follows: (1) duplicate publications; (2) interim or extension findings of the same trial, or with duplicate patients; (3) the number of included patients were less than ten; (4) retrospective studies.

### Data collection

Data were extracted from all eligible articles using standardized excel forms. Data retrieved from the articles included: (1) basic information of studies: first author name, publication year, study location, study design, number of participants, treatment and study phase, study primary and secondary outcomes and lung function inclusion criteria; (2) methodological qualities of the trials; (3) baselines and follow-up data of efficacy: renal angiomylipoma response rates, renal angiomylipoma volumes, 6-min walking distances, serum vascular endothelial growth factor D (VEGF-D) levels, forced expiratory volume in 1 s (FEV_1_), forced vital capacity (FVC) and the diffusing capacity for carbon monoxide (DL_CO_) values; (4) adverse events (AEs) (mentioned in at least 3 articles): event types and numbers of patients having all grade AEs.

### Quality assessment

The Cochrane criteria was used to systemically assess the bias in the RCTs, with the following items: adequacy of random sequence generation, allocation concealment, blinding of participants, personnel and outcome assessment, addressing of drop-outs or incomplete outcome data, selective outcome reporting, and other potential sources of bias. According to the methodological index for non-randomized studies (MINORS) [16], the qualities of single-arm trials were assessed, including eight items: a clearly stated aim, inclusion of consecutive patients, prospective data collection, endpoints appropriate to the aim of the study, unbiased assessment of the study endpoint, follow-up period appropriate to the study aim, loss to follow up less than 5%, prospective calculation of the study size.

### Data synthesis and statistical analysis

Data analysis was performed on the statistical software package *R* and Review Manager (*version 5.3*). We retrieved the mean and standard deviations (SD) values of continuous data from the included articles, such as serum VEGF-D levels, 6-min walk distances and the absolute values of FEV_1_, FVC and DL_CO_ at baseline and end-points. If the outcome measures were reported in the mean value and 95% confidence interval (95% CI), the SD values were estimated using the method in Review Manager calculator. The mean and SD values of FEV_1_ and FVC index [17] not reported in the full-text were estimated by the methods described in Cochrane handbook [18, 19]. For studies providing the raw individual data, baselines and endpoints values were calculated using *R*.

For single-arm clinical trials, net changes in continuous measurements were calculated as measure at endpoint of follow-up− measure at baseline. For RCTs, net changes were derived by (measure at end of follow-up in the treatment group− measure at baseline in the treatment group)−(measure at end of follow-up in the control group−measure at baseline in the control group). The random-effect model was used to explore the effect sizes. Heterogeneity was quantitatively assessed by *χ*^2^ test and *I*^*2*^ index(low heterogeneity:*I*^*2*^ ≤ 25%; moderate: 25–50%; high> 75%). The effect sizes were reported as weighted mean difference (WMD) and 95% CI. To analyze the robustness of the results, sensitivity analyses were processed by the leave-one-out method.

For dichotomous parameters, the pooled proportions were analyzed in random- effect model by *R*.

### Publication bias

The number of studies (less than 10) was low in the meta-analysis, publication bias could not be explored by funnel plot or Begg’s tests.

## Results

### Study selection and characteristics of included studies

**Fig. 1:**
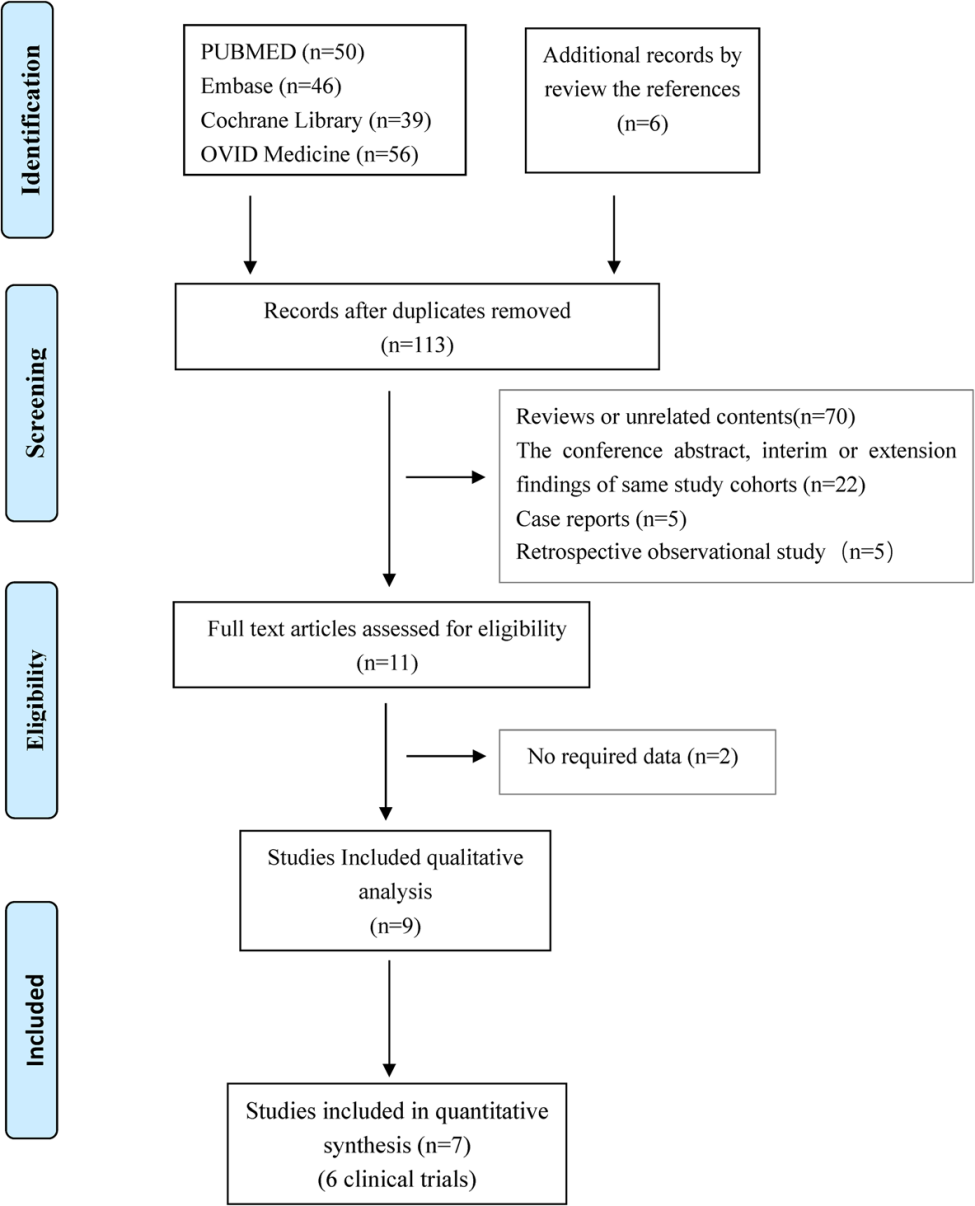
Study selection flow diagram

The process of searching and identifying studies was reported in Fig. 1. The eligibilities were carefully assessed in 11 full-text articles. Finally, only 9 studies met the inclusion criteria for the systematic review, including 2 RCTs [14, 20, 21], 6 single-arm trials [12, 17, 22–25], of which 7 studies were used for the meta-analysis. Notably, Budde et al. [21] reported the accurate data on the serum VEGF-D levels, which were not elaborated in the initial study [20], so we cited the two separate studies as the same RCT. We could not achieve the FEV_1_, FVC and DL_CO_ values at baselines and end-points from Takada et al. [24], Bissler et al. [20] and Bee et al. [25], so related results were only used for qualitative analyses.

Table 1 summarized the characteristics of the included studies. 211 subjects were included in the single arm trials, 125 patients in the RCTs treatment arms, and 82 patients in the RCTs placebo groups. The number of LAM patients was 300, accounting for 72% of the total population. In all studies, the diagnosis of TSC or LAM was confirmed. The quality evaluations of the trials were presented in Additional file 1 Table S1 and Table S2.

**Table 1:** Characteristics of included studies in the systematic review and meta-analysis

Author, Publication Year, Country	Journal	Study design	No of patients^a^	Study duration	Intervention and sample size	Study outcomes	Lung function inclusion criteria
Bissler[11], 2008, USA	N Engl J Med	single-center prospective open-label phase 1–2 trial	25 (6/12/7)	treatment 1 year, observation 1 year	Sirolimus (*n* = 25)	Renal AMLs, lung function, 6MWD, lung-cyst volume, AEs and neurologic assessment.	NA
Davies [21], 2011, UK	Clin Cancer Res	multicenter prospective nonrandomized open label phase 2 trial	16 (6/3/7)	treatment 2 years	Sirolimus (*n* = 16)	Renal AMLs, lung function, AEs and neurocognitive function.	NA
McCormack [14], 2011, USA	N Engl J Med	multi-center, randomized, placebo-controlled study (MILES)	89 (81/8/0)	treatment 1 year, obervation 1 year	sirolimus group (*n* = 46); placebo group (*n* = 43)	Lung function, 6MWD, VEGF-D levels and QOL scores and AEs.	FEV_1_ ≤ 70%
Dabora [22], 2011,USA	PLoS ONE	multicenter, open label, single arm, phase 2 trail	36 (0/21/15)	treatment 1 year	sirolimus (*n* = 36)	Renal AMLs, lung function, brain tumors and liver AMLs, skin lesions, VEGF-D levels, AEs.	NA
Bissler [13],2013, USA	Lancet	multicenter randomized, double-blind, placebo-controlled, phase 3 study (EXIST-2)	118 (5/24/89)	median everolimus 38 weeks (10–85 weeks)	everolimus group (*n* = 79); placebo group (*n* = 39)	AMLs, skin lesion, pulmonary function, VEGF-D levels, everolimus pharmacokinetics and AEs.	DL_CO_ > 35%
Budde [20], 2015, USA	Brit J Clin Pharmaco	multicenter randomized, double-blind, placebo-controlled, phase 3 study (EXIST-2)	79 (2/20/57)	median everolimus 38 weeks (10–85 weeks)	everolimus(*n* = 79)	Associations between everolimus concentration and AMLs size, VEGF-D and other biomarker levels.	DL_CO_ > 35%
Goldberg [17], 2015, USA	Eur Respir J	multicenter, open-label, nonrandomised, phase 2 trial	24 (19/5/0)	26 weeks	everolimus, (*n* = 24)	AEs, everolimus pharmacokinetics, VEGF-D levels, lung function.	(30% ≤ FEV_1_ ≤ 80%) or (30% ≤ FEV_1_ ≤ 90% and DL_CO_ < 80%)
Takada [23], 2016, Japan	Annals ATS	mulcenter single arm, open-label trial (MLSTS)	63^b^	2-year study period	Sirolimus (*n* = 63)	AEs, lung function and QOL scores.	NA
Bee [24], 2017, UK	Thorax	a prospective national cohort study, single arm	47 (38/9/0)	35.8 ± 18 months	Sirolimus (*n* = 47)	Lung function, AEs.	NA

*AMLs* angiomyolipomas, *AEs* adverse events, *DL*_*CO*_ diffusing capacity for carbon monoxide, *FEV*_*1*_ forced expiratory volume in 1 s, *LAM* lymphangioleiomyomatosis, *QOL* quality of life, *TSC* tuberous sclerosis complex, *VEGF-D* vascular endothelial growth factor D, *RV* residual volume, *6MWD* 6-min walking distance

^a^ represent the number of subjects with the diagnosis of sporadic LAM(sLAM), TSC-LAM, and only TSC without LAM manifestation

^b^LAM patients were included in the study, no accurate data on the number of sLAM or TSC-LAM

### Effects on lung function, 6-min walking tests and quality of life scores

**Fig. 2:**
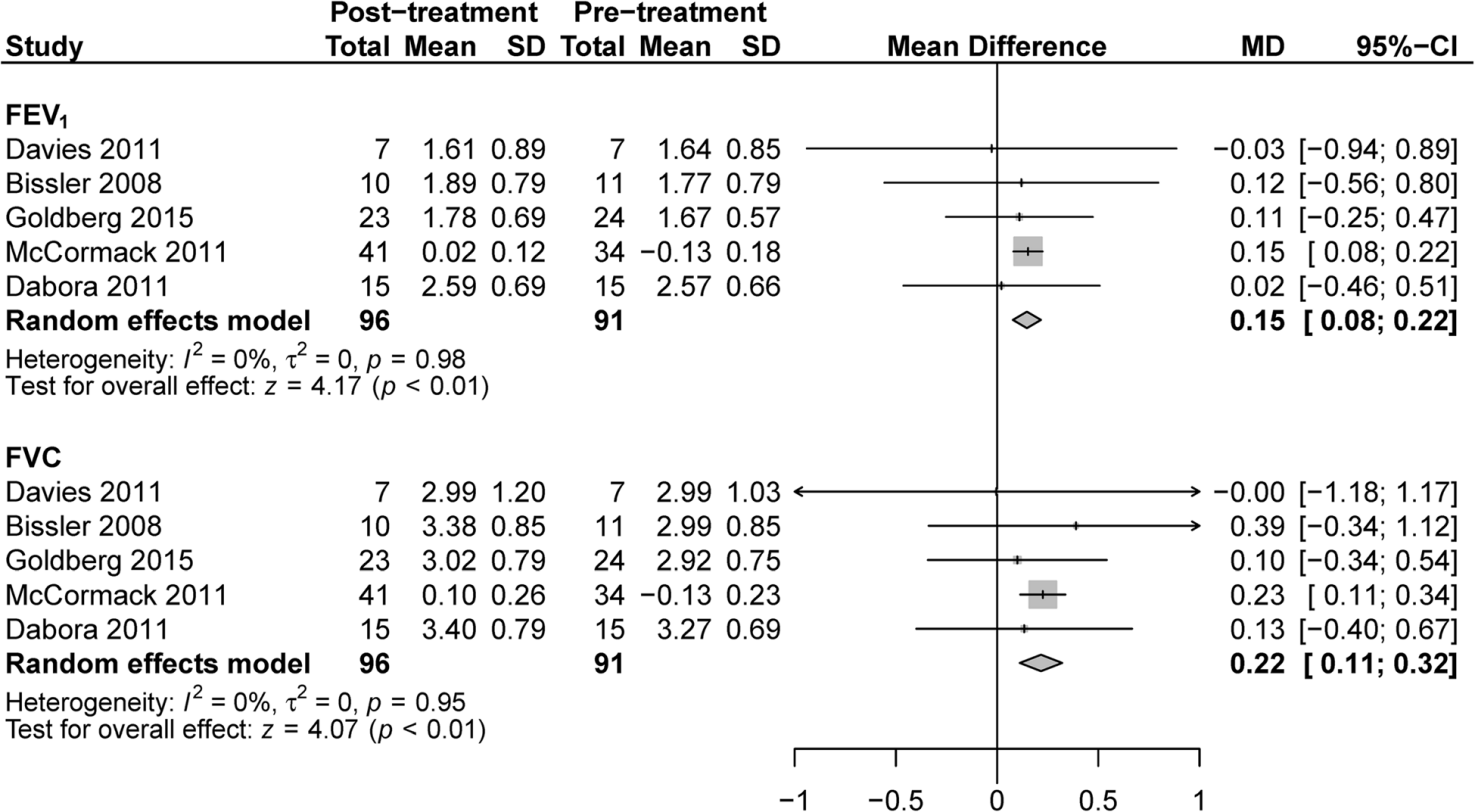
Forest plot for the weighted mean difference of FEV_1_ and FVC with 95% confidence interval in the random effects model

The FEV_1_ and FVC values showed a significant increase after therapy, with the WMD 0.15 L (95%CI: 0.08 to 0.22, *P* < 0.01, *I*^*2*^ = 0%, Fig. 2) and 0.22 L (95%: 0.11 to 0.32, *P* < 0.01, *I*^*2*^ = 0%, Fig. 2) respectively. Neither the DL_CO_ (WMD: 0.51 ml/mm Hg/min, 95%CI: -0.48 to 1.49, *P* = 0.31, *I*^*2*^ = 0%, Additional file 1 Figure S1) nor 6-min walking distance (WMD: 5.29 m, 95%CI: -18.01 to 28.59, *P* = 0.66, *I*^*2*^ = 1%, Additional file 1 Figure S2) changed significantly. The pulmonary functions and 6-min walking tests were all conducted in LAM patients in the included trials, with 26 weeks [17] or 1 year [12, 14, 22, 23] treatment periods.

Additionally, only 2 studies evaluated the therapy effects on EuroQOL visual-analogue scale (VAS) scores and Functional Performance Inventory (FPI) total score among LAM patients. MILES trail found significant improvements of score changes on VAS and FPI in sirolimus group compared with changes in placebo group [17]. On the other hand, Takada et al. [17] found no enhance on PFI and VAS score after sirolimus therapy in treated LAM patients.

### Effects on renal angiomyolipoma volume and VEGF-D levels

**Fig. 3:**
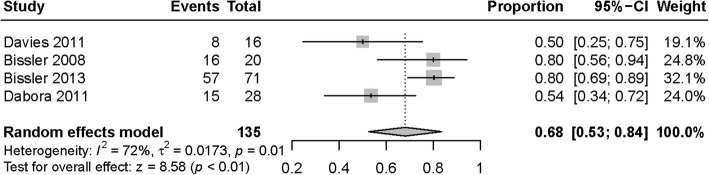
Forest plot for the weighted-pooled proportion of renal angiomyolipoma partial response with 95% confidence interval in the random effects model

**Fig. 4:**
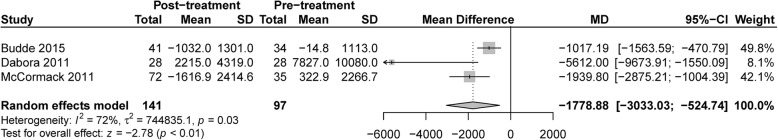
Forest plot for the weighted mean difference of VEGF-D levels with 95% confidence interval in the random effects model

The evaluation on the renal angiomyolipoma response rate was the primary outcome in 4 studies [12, 20, 22, 23]. The weighted partial response rate was 0.68 (95%CI: 0.53 to 0.84, *P* < 0.01, *I*^*2*^ = 72%, Fig. 3) in random-effect model, far higher than the reported proportion (0.03(1/33)) in EXIST-2 control group [20]. Three studies measured the levels of VEGF-D before and after therapy, including 2 RCTs [14, 21] and 1 single-arm trail [23]. The weighted mean difference was − 1778.88 ng/ml (95%CI: -3033.03 to − 524.74, *P* < 0.01, *I*^*2*^ = 72%, Fig. 4).

### Safety events of mTOR inhibitors in treated LAM patients

**Fig. 5:**
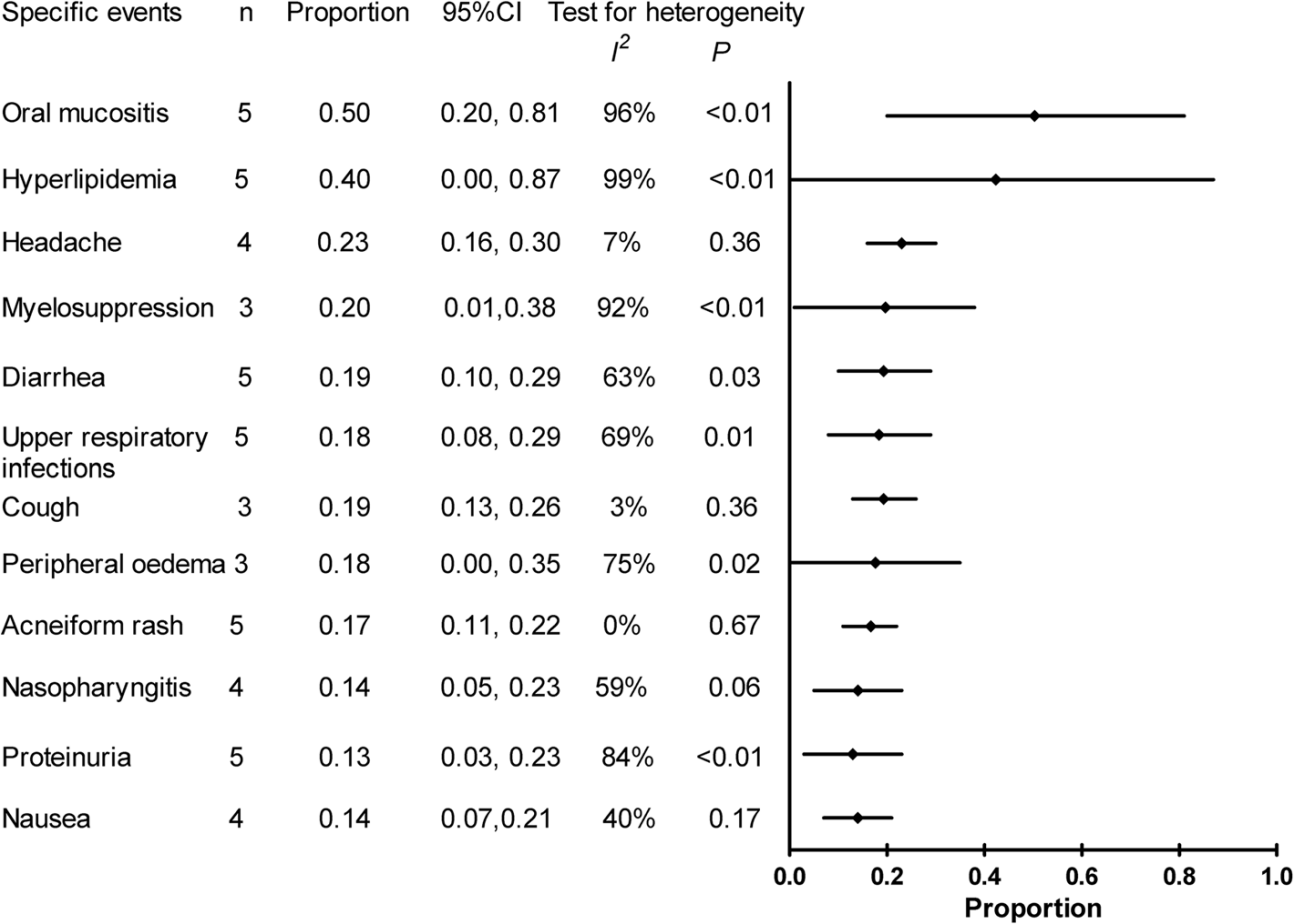
Forest plot for the weighted-pooled proportions of adverse events in patients receiving sirolimus or everolimus including the 95% confidence interval from random effect model and number of included study(n)

Data regarding to the number of patients occurring specific safety events were available in 5 studies [12, 17, 20, 22, 23]. The safety outcomes in McCormack et al. [14], Takada et al. [24] and Bee et al. [25] were not included because of no access to the number of patients. The common AEs during therapy were oral mucositis (50%), hyperlipidemia (40%), headache (23%), bone marrow suppression (20%), diarrhea (19%) and cough (19%), followed with upper respiratory tract infections (18%), peripheral oedema (18%), acneiform rash (17%), nasopharyngitis (14%), nausea (14%) and proteinuria (13%) (Fig. 5). And most events were low grade and tolerant. Nine pneumonitis were identified in 5 trials [12, 17, 20, 22–24].

After excluding the results from MILES [14], net changes for FEV_1_ turned to be insignificant (WMD: 0.08 L, 95%CI: -0.18 to 0.33, *P* = 0.56, *I*^*2*^ = 0%), the same went for FVC (WMD: 0.15 L, 95%CI: -0.15 to 0.45, *P* = 0.32, *I*^*2*^ = 0%). The sensitivity analyses indicated that data from the study conducted by Dabora et al. [23] was the main source of heterogeneity in the analyses of adverse events. Omitting the study of Dabora et al. [23], *I*^*2*^ index decreased to 54, 72.7, 28.4, 45 and 11.7% for oral mucositis, hyperlipidemia, bone marrow suppression, proteinuria, and peripheral oedema respectively.

## Discussion

The quantitative analysis supported that significant improvements in the pulmonary function (FEV_1_ and FVC) and alleviations of the AML burden could be obtained after the application of mTOR inhibitors in LAM patients. However, with respect to DL_CO_ and 6-min walking distance, our results indicated insignificant changes after the therapy. The proportions of adverse events in LAM patients were quantified, and the common toxicity events during the mTOR inhibitors therapy were oral mucositis, hyperlipidemia, headache, bone marrow suppression, diarrhea and cough.

Previous studies had shown that the impairment of lung function in LAM may be caused by the remodeling in the airway and lung parenchyma, which were associated with the irrational LAM cell infiltration [26] or the tissue damage from the matrix metalloproteinases released by LAM cells [27]. Desirably, mTOR inhibitors could inhibit the cell proliferation and reduce tumor cell volume. As a consequence, the LAM cell burden could be alleviated and the progressive airflow obstruction could be stabilized. The quantitative computed tomographic analysis further revealed the protective effect of mTOR inhibitors in LAM from imaging modalities [28].

To our knowledge, no meta-analysis has ever evaluated the therapeutic effects of mTOR inhibitors in LAM patients. Our quantitative analyses indicated the improvements of FEV_1_ and FVC after therapy, which were consistent with the trends in previous studies [12, 14, 17, 23, 25, 29]. Nevertheless, the effect sizes of FEV_1_ and FVC turned into insignificant when the control groups [14] were excluded in this meta-analysis. Several analyses ever found no increment after the mTOR inhibitors therapy, all without comparisons with the placebo group [20, 22, 30]. So, the roles of controlled group stood out. The administration of mTOR inhibitors in LAM patients can achieve increment and stability for FEV_1_ and FVC compared with the untreated patients. The control groups are recommended for accurate and comprehensive understanding in further clinical trials.

Other benefits from therapy of mTOR inhibitors in LAM subjects were the shrinkage of renal angiomylipoma volume and statistically decrease of VEGF-D levels. In our analysis, the partial response rate was 68%. In the 4-year extension therapy [30], the response rate could be maintained among over 50% patients, and the number of patients achieving over 50% reductions increased over time. Previous studies [12, 14] had suggested that the treatment relief in angiomylipoma volume and lung function tended to reverse after the drug withdrawn, though not with accelerating bounce. Hence, maintenance therapy would be benefited for LAM patients conforming to the therapy indications. However, clinical trials included in our meta-analysis didn’t evaluate the therapy effect of sirolimus on recurrent pneumothorax and chylothorax, which may limit the evaluation on lung function due to restrictive ventilation dysfunction. Previous systematic review summarized common measures for treating chylothorax among LAM patients and observed that sirolimus therapy obtained largest favorable outcomes [31]. Nevertheless, those results needed to be interpreted with caution, because of different therapy combination and short follow-up periods.

Further, the pooled cumulative incidence rates of the safety events were calculated in this meta-analysis based on existing reports. The common events were similar in treated arms, and most reported adverse events were tolerable and low-grade in severity. Because only one RCT trial [20] fulfilled our safety analysis criteria, the odds ratios were unable to assess convincingly. One study suggested the treatment of mTOR inhibitors did not increase the incidence of respiratory infections, with assumption that the incidence rate was constant [32]. While several studies reported that the occurrence of adverse events tended to reduce over time during the mTOR inhibitors therapy [24, 25, 30]. Therefore, long-term follow-up and multicenter trials with control group are needed to better evaluate the balance of benefits and risks in LAM patients.

Our study had several limitations. Firstly, only one RCT was included in the quantitative analysis due to lack of raw data. Secondly, four studies including TSC patients, which may confound the evaluations on safety events and AML response rate. Finally, the considerable data extracted from included studies was unavailable for subgroup analyses to assess the influences of certain variables.

## Conclusions

In summary, our systematic review and meta-analysis provided quantitative and updated supports for the administration of mTOR inhibitors in LAM patients, especially in improving the pulmonary function and alleviating renal angiomylipoma. Our systematic review also suggested that LAM patients received long-term mTOR inhibitors therapy showed decreased occurrence of tolerable AEs and the continuous improvements of pulmonary functions and renal angiomylipoma volumes. Further studies with long-term follow-up are warranted to establish the long-term benefits, to supervise the safety events and to recognize disease phenotypes according to the disease trait and therapy response.

## Additional file

Additional file 1: **Table S1.** Quality assessment for randomized controlled trials by Cochrane risk evaluation tool. **Table S2.** Quality assessment for single-arm trials by the methodological index for non-randomized studies (MINORS) criteria. **Figure S1.** Forest plot for the weighted mean difference of DL_CO_ with 95% confidence interval in the random effects model. **Figure S2.** Forest plot for the weighted mean difference of 6-min walking distance with 95% confidence interval in the random effects model. (DOCX 139 kb)

## Abbreviations

6MWD: 6-min walking distance
AEs: Adverse events
AMLs: Renal angiomyolipomas;
CI: Confidence interval
DL_CO_: Diffusing capacity for carbon monoxide
FEV_1_: Forced expiratory volume in 1 s
FVC: Forced vital capacity
LAM: Lymphangioleiomyomatosis
mTOR: Mammalian target of the rapamycin
SD: Standard deviations
sLAM: Sporadical lymphangioleiomyomatosis
TSC: Tuberous sclerosis complex
VEGF-D: Vascular endothelial growth factor D
WMD: Weighted mean difference

## References

[1] Ryu JH, Moss J, Beck GJ (2006). The NHLBI lymphangioleiomyomatosis registry: characteristics of 230 patients at enrollment. Am J Respir Crit Care Med.

[2] Travis WD, Brambilla E, Nicholson AG (2015). The 2015 World Health Organization classification of lung tumors: impact of genetic, clinical and radiologic advances since the 2004 classification. J Thorac Oncol.

[3] McCormack FX (2008). Lymphangioleiomyomatosis: a clinical update. Chest.

[4] Sengupta S, Peterson TR, Sabatini DM (2010). Regulation of the mTOR complex 1 pathway by nutrients, growth factors, and stress. Mol Cell.

[5] Yu J, Astrinidis A, Henske EP (2001). Chromosome 16 loss of heterozygosity in tuberous sclerosis and sporadic lymphangiomyomatosis. Am J Respir Crit Care Med.

[6] Henske EP (2003). Metastasis of benign tumor cells in tuberous sclerosis complex. Genes Chromosomes Cancer.

[7] Seyama K, Kumasaka T, Kurihara M, Mitani K, Sato T (2010). Lymphangioleiomyomatosis: a disease involving the lymphatic system. Lymphat Res Biol.

[8] Harknett EC, Chang WY, Byrnes S (2011). Use of variability in national and regional data to estimate the prevalence of lymphangioleiomyomatosis. QJM.

[9] Crino PB, Nathanson KL, Henske EP (2006). The tuberous sclerosis complex. N Engl J Med.

[10] Taveira-DaSilva AM, Stylianou MP, Hedin CJ, Hathaway O, Moss J (2004). Decline in lung function in patients with lymphangioleiomyomatosis treated with or without progesterone. Chest.

[11] Johnson SR, Tattersfield AE (1999). Decline in lung function in lymphangioleiomyomatosis: relation to menopause and progesterone treatment. Am J Respir Crit Care Med.

[12] Bissler JJ, McCormack FX, Young LR (2008). Sirolimus for angiomyolipoma in tuberous sclerosis complex or lymphangioleiomyomatosis. New England Journal of MedicineNew Engl J Med.

[13] Davies DM, Johnson SR, Tattersfield AE (2008). Sirolimus therapy in tuberous sclerosis or sporadic lymphangioleiomyomatosis. New England Journal of MedicineNew Engl. J. Med...

[14] McCormack FX, Inoue Y, Moss J (2011). Efficacy and safety of sirolimus in lymphangioleiomyomatosis. N Engl J Med.

[15] McCormack FX, Gupta N, Finlay GR (2016). Official American Thoracic Society/Japanese respiratory society clinical practice guidelines: Lymphangioleiomyomatosis diagnosis and management. Am J Respir Crit Care Med.

[16] Slim K, Nini E, Forestier D, Kwiatkowski F, Panis Y, Chipponi J (2003). Methodological index for non-randomized studies (minors): development and validation of a new instrument. ANZ J Surg.

[17] Goldberg HJ, Harari S, Cottin V (2015). Everolimus for the treatment of lymphangioleiomyomatosis: a phase II study. Eur Respir J.

[18] Furukawa TA, Barbui C, Cipriani A, Brambilla P, Watanabe N (2006). Imputing missing standard deviations in meta-analyses can provide accurate results. J Clin Epidemiol.

[19] Higgins JPT DJJ, Collaboration ADG. (TC. Chapter 16: Special topics in statistics. In: Higgins JPT, Green S (editors). Cochrane Handbook for Systematic Reviews of interventions. Version 5.0.1. 2008.

[20] Bissler JJ, Kingswood JC, Radzikowska E (2013). Everolimus for angiomyolipoma associated with tuberous sclerosis complex or sporadic lymphangioleiomyomatosis (EXIST-2): a multicentre, randomised, double-blind, placebo-controlled trial. Lancet.

[21] Budde K, Zonnenberg BA, Frost M (2015). Pharmacokinetics and pharmacodynamics of everolimus in patients with renal angiomyolipoma and tuberous sclerosis complex or lymphangioleiomyomatosis. Br J Clin Pharmacol.

[22] Davies DM, de Vries PJ, Johnson SR (2011). Sirolimus therapy for angiomyolipoma in tuberous sclerosis and sporadic lymphangioleiomyomatosis: a phase 2 trial. Clin Cancer Res.

[23] Dabora SL, Franz DN, Ashwal S (2011). Multicenter phase 2 trial of sirolimus for tuberous sclerosis: kidney angiomyolipomas and other tumors regress and VEGF- D levels decrease. PLoS One.

[24] Takada T, Mikami A, Kitamura N (2016). Efficacy and safety of long-term Sirolimus therapy for Asian patients with Lymphangioleiomyomatosis. Ann Am Thorac Soc.

[25] Bee J, Fuller S, Miller S, Johnson SR. Lung function response and side effects to rapamycin for lymphangioleiomyomatosis: a prospective national cohort study. Thorax. 2018;73(4):369–75. 10.1136/thoraxjnl-2017-210872 28993539

[26] Corrin B, Liebow AA, Friedman PJ (1975). Pulmonary lymphangiomyomatosis. A review Am J Pathol.

[27] Chilosi M, Pea M, Martignoni G, Brunelli M, Gobbo S, Poletti V, Bonetti F. Cathepsin-k expression in pulmonary lymphangioleiomyomatosis. Mod Pathol. 2009;22(2):161–6. 10.1038/modpathol.2008.189 19060845

[28] Argula RG, Karwa A, Lauer A, Gregg D, Silver RM, Feghali-Bostwick C, Schanpp LM, Egbert K, Usher BW, Ramakrishnan V, Hassoun PM, Strange C. A Novel quantitative computed tomographic analysis suggests how Sirolimus stabilizes progressive air trapping in Lymphangioleiomyomatosis. Ann Am Thorac Soc. 2017;13(3):342–9. 10.1513/AnnalsATS.201509-631OC PMC5015717 26799509

[29] Zhan Y, Shen L, Xu W (2018). Functional improvements in patients with lymphangioleiomyomatosis after sirolimus: an observational study. Orphanet J Rare Dis.

[30] Bissler JJ, Kingswood JC, Radzikowska E (2017). Everolimus long-term use in patients with tuberous sclerosis complex: four-year update of the EXIST-2 study. PLoS One.

[31] Lama A, Ferreiro L, Golpe A, Gude F, Álvarez-Dobaño JM, González-Barcala FJ, Toubes ME, San JE, Rodríguez-Núñez N, Valdés L (2016). Characteristics of patients with Lymphangioleiomyomatosis and pleural effusion: a systematic review. Respiration.

[32] Courtwright AM, Goldberg HJ, Henske EP, El-Chemaly S. The effect of mTOR inhibitors on respiratory infections in lymphangioleiomyomatosis. Eur Respir Rev. 2017;26(143). 10.1183/16000617.0004-2016 PMC9484491 28096282

