# Supplementary material for: The efficacy and adverse events of mTOR inhibitors in lymphangioleiomyomatosis: systematic review and meta-analysis
Source: Orphanet J Rare Dis. 2018 Aug 14;13:134. doi: 10.1186/s13023-018-0874-7 (PMC6092843; doi:10.1186/s13023-018-0874-7)
Supplement: Supplementary file 1 — Table S1. Quality assessment for randomized controlled trials by Cochrane risk evaluation tool. Table S2. Quality assessment for single-arm trials by the methodological index for non-randomized studies (MINORS) criteria. Figure S1. Forest plot for the weighted mean difference of DLCO with 95% confidence interval in the random effects model. Figure S2. Forest plot for the weighted mean difference of 6-min walking distance with 95% confidence interval in the random effects model. (DOCX 139 kb) [file 13023_2018_874_MOESM1_ESM.docx]

**Supplement**

**The efficacy and adverse events of mTOR inhibitors in lymphangioleiomyomatosis: systematic review and meta-analysis**

Nannan Gao^1^, Tengyue Zhang^1^, Jiadong Ji^2^, Kaifeng Xu^1^, Xinlun Tian^1*^

^1^Department of Respiratory Medicine, Peking Union Medical College Hospital, Peking Union Medical College, Chinese Academy of Medical Sciences, Beijing, China.

^2^School of Statistics, Shandong University of Finance and Economics, Jinan, China.

**^*^Corresponding author**: Xinlun Tian, e-mail: xinlun_t@sina.com

| Author, Publication year | adequacy of random sequence generation | allocation concealment | blinding of participants | personnel and outcome assessment | addressing of drop-outs | selective outcome reporting |
| --- | --- | --- | --- | --- | --- | --- |
| McCormack,2011 | Unclear | low risk of bias | low risk of bias | low risk of bias | low risk of bias | low risk of bias |
| Bissler,2013 | Unclear | unclear | low risk of bias | low risk of bias | low risk of bias | high of risk |

Table S1 Quality assessment for randomized controlled trials by Cochrane risk evaluation tool.

Table S2 Quality assessment for single-arm trials by the methodological index for non-randomized studies (MINORS) criteria.

| Author, publication year | a clearly stated aim | | | inclusion of consecutive patients | prospective data collection | endpoints appropriate to the aim of the study | unbiased assessment of the study endpoint | | follow-up period appropriate to the study aim | | loss to follow up less than 5% | prospective calculation of the study size | |
| --- | --- | --- | --- | --- | --- | --- | --- | --- | --- | --- | --- | --- | --- |
| Davies,2011 | 2 | | | 2 | 2 | 2 | 0 | | 2 | 0 | | | 2 |
| Bissler,2008 | 2 | | | 2 | 2 | 2 | 0 | | 2 | 0 | | | 2 |
| Goldberg,2015 | | | 2 | 2 | 2 | 2 | 0 | | 2 | 0 | | | 2 |
| Dabora,2011 | | 2 | | 2 | 2 | 2 | 0 | | 2 | 0 | | | 2 |
| Takada,2016 | | 2 | | 2 | 2 | 2 | 0 | | 2 | 0 | | | 0 |
| Bee, 2017 | 2 | | | 2 | 2 | 2 | 0 | 2 | | | 2 | | 0 |

0 score: not reported; 1 score: reported with inadequate information; 2 scores: reported with adequate information.


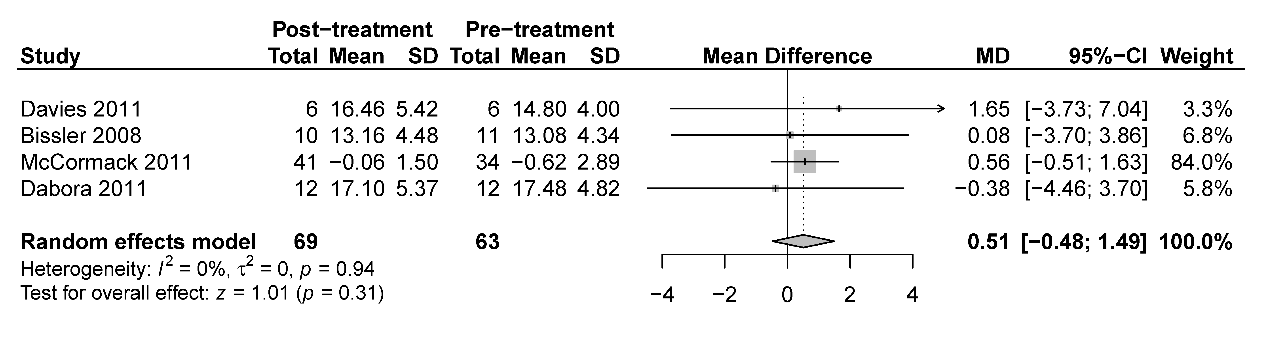


Figure S1 Forest plot for the weighted mean difference of DL_CO_ with 95% confidence interval in the random effects model.


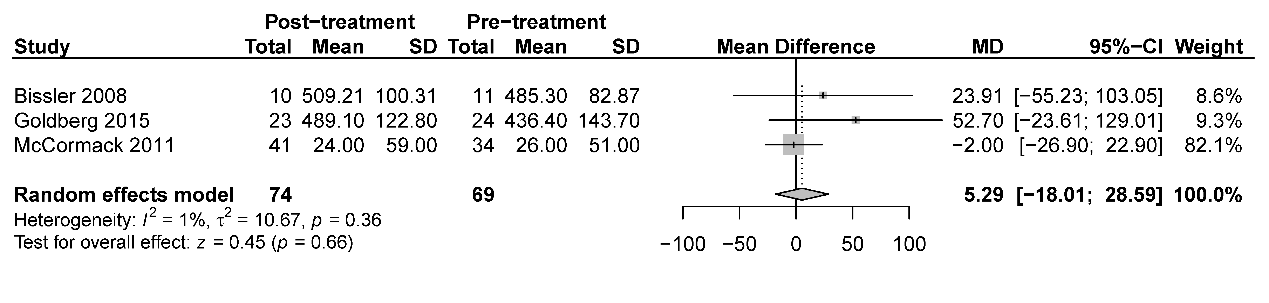


Figure S2 Forest plot for the weighted mean difference of 6-minute walking distance with 95% confidence interval in the random effects model.
